# Laboratory Guide for the Bacteriologist

**Published:** 1895-04

**Authors:** 


LABORATORY GUIDE FOR THE BACTERIOLOGIST. By Langdon Frothingham, M. D. V., Assistant in Bacteriology and Veterinary Science, Sheffield Scientific School, Yale University. Illustrated. Price, 75c. Philadelphia: W. B. Saunders, 925 Walnut Street. 1895.

For the practical study of bacteriology in the laboratory, this concise and well arranged scheme will be a great aid to students in their early work, and to experienced bacteriologists as a ready reference guide in staning, preparation of sections, etc. The author simply intends his book to be used as a convenient reference, taking the place of larger works of this class. V. H. T.

